# Comparing Veterans Preferences and Barriers for Video Visit Utilization Versus In-Person Visits: a Survey of Two VA Centers

**DOI:** 10.1007/s11606-023-08494-9

**Published:** 2024-01-22

**Authors:** Omar El-Shahawy, Andrew Nicholson, Nicholas Illenberger, Lisa Altshuler, Anne Dembitzer, Paul Krebs, Melanie Jay

**Affiliations:** 1 Population Health Department, New York University Grossman School of Medicine, New York, NY USA; 2 Global and Environmental Health Department, New York University School of Global Public Health, New York, NY USA; 3 Veterans Affairs New York Harbor Healthcare System, New York, NY USA; 4 Department of Medicine, New York University School of Medicine, New York, NY USA

## INTRODUCTION

With the onset of COVID, the VA like other health care systems in the USA and globally had to rapidly expand their telehealth services.^1^ Prior to COVID-19, the Veterans Health Administration (VA) offered telehealth services, predominantly focused on Veterans with healthcare access barriers.^2^

There are limited studies, with mixed evidence, evaluating patients’ preferences for video visits compared with in-person visits.^3, 4^ To address this gap, we conducted a quality improvement survey between March and August 2021 among Veterans to evaluate their experiences with video visit utilization during the first year of COVID-19.

## METHODS

We evaluated Veterans’ experiences with video versus in-person using six items from prior validated surveys.^5^ We used the national VA Corporate Data Warehouse to identify all patients who had ≥ 1 primary care clinical video visit from 3/2/2020 to 12/31/2020 at either San Diego or VA NY Harbor. We mailed 2208 unique Veterans the survey from May to October 2021 and received 493 survey responses. We excluded *n* = 93 surveys for never having a video visit or missing outcome variables, with a final analytical sample of *N* = 400, and adjusted response rate of 24.5%, with over half the sample from NY Harbor VA (*n* = 206, 51.5%).

We categorized individuals into two preference groups based on scored responses to a 6-item survey on visit modality preference. Scores ranged from 33 to 100 (mean 50.4, SD 16.2) with higher scores indicating a preference for video visits. *Video pessimists* scored below-mean in video visit preference compared to in-person visits, and *video enthusiasts* had above-mean video visit preference compared to in-person visits. We assessed Veterans’ characteristics associated with visit preference using logistic regression. Reported barriers for video visit utilization were compared using the chi-square or Fisher’s exact test using SAS version 9.4 and *p* < 0.05.

## RESULTS

A total of 400 Veterans were surveyed. 41% preferred video compared to in-person visits. Table 1 presents the characteristics of the total sample and findings of the adjusted logistic regression model. Having internet access most of the time vs occasionally/never was associated with video visit preference (AOR = 3.6; 95%CI: 1.6, 8.2); also, older Veterans (71 + years) were less likely to be video enthusiasts compared to younger Veterans (AOR = 0.4; 95%CI: 0.2, 0.8). Barriers for video utilization are shown in Table 2.

**Table 1:** Participant Characteristics and Factors Associated with Video Visit Preference Group (*n* = 400)

	Total sample *N* = 400	Video pessimists^a^ *n* = 236	Video enthusiasts^b^ *n* = 164	Video enthusiasts (regression model results)	
	*n* (%)	*n* (%)	*n* (%)	Odds ratio (95% CI)	*p*-value (regression)
Age					
25–50	87 (21.8)	35 (14.8)	52 (31.7)	1.00	
51–70	166 (41.5)	95 (40.3)	71 (43.3)	0.7 (0.4, 1.3)	0.30
71 +	147 (36.8)	106 (44.9)	41 (25.0)	**0.4 (0.2, 0.8)**	0.01*
Sex					
Male	325 (81.25)	207 (87.7)	118 (71.9)	1.00	
Female	75 (18.75)	29 (12.3)	46 (28.1)	1.6 (0.9, 2.9)	0.11
Race and ethnicity					
NH White	175 (43.8)	103 (43.6)	72 (43.9)	1.00	
NH Black	73 (18.3)	43 (18.2)	30 (18.3)	0.8 (0.4, 1.5)	0.47
Hispanic	86 (21.5)	46 (19.5)	40 (24.4)	1.1 (0.6, 1.9)	0.84
NH other^c^	66 (16.5)	44 (18.6)	22 (13.4)	0.6 (0.3, 1.1)	0.09
Education					
Some college or less	200 (50.0)	131 (55.5)	69 (42.1)	1.00	
Bachelor or higher	200 (50.0)	105 (44.5)	95 (57.9)	1.2 (0.8, 1.9)	0.45
Employment					
Employed	124 (31.0)	55 (23.3)	69 (42.1)	1.00	
Retired	152 (38.0)	102 (43.2)	50 (30.5)	0.9 (0.5, 1.8)	0.83
Unemployed/other^d^	124 (31.0)	79 (33.5)	45 (27.4)	0.8 (0.5, 1.5)	0.57
Household income					
Up to $40,000	104 (26.0)	71 (30.1)	33 (20.1)	1.00	
≥ $40,000	201 (50.3)	99 (42.0)	102 (62.2)	1.6 (0.9, 2.9)	0.10
Prefer not to answer	95 (23.8)	66 (28.0)	29 (17.7)	1.0 (0.5, 1.8)	0.96
Married/living with partner					
No	166 (41.5)	143 (60.6)	73 (44.5)	1.00	
Yes	234 (58.5)	93 (39.4)	91 (55.5)	0.9 (0.5, 1.4)	0.53
Pre-COVID video experience					
No	171 (42.75)	100 (42.4)	71 (43.3)	1.00	
Yes	229 (57.25)	136 (57.6)	93 (56.7)	0.8 (0.5, 1.3)	0.37
Internet accessibility					
Occasionally/never	54 (13.5)	46 (19.5)	8 (4.9)	1.00	
Most of the time	346 (86.5)	190 (80.5)	156 (95.1)	**3.6 (1.6, 8.2)**	< 0.01*

*p*-values are presented for logistic regression of the factors associated with visit preference group. Significant results are highlighted in bold text. The reference group is video pessimists (i.e., respondents who scored at or below the mean rating for video visit preference using 6 items comparing patient experience between video and in-person visit). The 6 items were “Overall quality of the visit”; “Personal connection I feel with clinician during the visit”; “Ability to show clinician a physical problem”; “Confidence health concern can be taken care of”; “Comfort I feel sharing personal or private information”; and “Amount of time I spend with my clinician” over a 5-point Likert scale

^a^*Video pessimists* scored at or below mean video visit preference compared to in-person visit

^b^*Video enthusiasts* scored above-mean video visit preference compared to in-person visit

^c^NH other group includes Asian, Pacific Islander/Native Hawaiian, multi-race, other, and prefer not to answer

^d^Other employment categories include student, disabled, and prefer not to answer

**Table 2:** Reported Barriers for Video Visit utilization by Group (*N* = 400)

Reported barriers (statements are noted in footnotes d–h)	Total	Video enthusiasts^a^	Video pessimists^b^	*p*-value^c^
	*N* = 400	*N* = 164	*N* = 236	
Concerns about the security and privacy of health information^d^				< 0.01
Yes (vs no)	41 (10.3)	8 (4.9)	33 (14.0)	
Discomfort with video visits^e^				< 0.01
Yes (vs no)	48 (12.0)	8 (4.9)	40 (16.9)	
The doctor’s inability to perform physical examination^f^				< .0001
Yes (vs no)	185 (46.3)	32 (19.5)	153 (64.8)	
Interference with patient’s everyday routine^g^				0.02
Yes (vs no)	29 (7.3)	6 (3.7)	23 (9.7)	

Responses for each reported barrier were “Strongly agree,” “Agree,” “Disagree,” “Strongly disagree,” and “Not applicable.” Barriers to video visit utilization were defined as “Yes” if the person responded strongly agree or agree and “No” if the person responded disagree or strongly disagree

^a^*Video enthusiasts* scored above-mean video visit preference compared to in-person visit

^b^*Video pessimists* scored at or below mean video visit preference compared to in-person visit

^c^*p*-values presented for the chi-square test

^d^Video visits made me worried about the security and privacy of my health information when being shared with my provider

^e^The video visits made me feel uncomfortable

^f^The doctor’s inability to perform physical examination discourages me to have a video visit in the future

^g^The video visits interfered with my everyday routine

## DISCUSSION

This study examined Veteran experiences with video visits during COVID-19. Less than half the sample preferred video to in-person visits, which is consistent with prior research.^4^ Similar to other studies, internet access was a potential disparity that influenced video telehealth preference. While concerns about confidentiality and discomfort with video utilization were main barriers early on during COVID-19,^5^ we found this concern in only 10% of Veterans upon conducting our survey in 2021. While inability to perform an examination remains a major concern for physicians during video visits, ^4^ around half of the Veterans continue to share this concern, albeit with significant variability between groups. Video pessimists were more likely to share this concern. They were also more likely to report that video visits interfered with their everyday routine, which may be due to their lower internet accessibility and comfort in use of technology. These data support the need for digital health literacy and access to technology to improve Veterans’ experiences with video telehealth.^6^

There are limitations to our study. Given its cross-sectional nature, we cannot make inferences about temporality and therefore causality. We cannot exclude the possibility of residual confounding. The analyses were based on self-reported data which are subject to misclassification, recall, and social desirability bias. Finally, our survey may not be generalizable to non-Veterans and patients from rural areas and areas outside the USA.

This study presents insightful findings on Veterans’ barriers to video visit utilization by their video preference. However, further qualitative studies exploring the preferences and concerns about telemedicine should guide future care. In conclusion, for the VA to ensure convenient, accessible, and patient-centered care, they may consider accounting for patient visit modality preferences and leverage patient-physician communication in telehealth platforms to ensure patient engagement and quality of care.^7^

## Data Availability

The de-identified data that support the findings of this study are available upon reasonable request from the corresponding author.

## References

[1] Chen PV, Helm A, Fletcher T, Wassef M, Hogan J, Amspoker A (2021). Seeing the value of video: a qualitative study on patient preference for using video in a Veteran Affairs Telemental Health program evaluation. Telemedicine reports..

[2] Zulman DM, Wong EP, Slightam C, Gregory A, Jacobs JC, Kimerling R (2019). Making connections: nationwide implementation of video telehealth tablets to address access barriers in Veterans. JAMIA open..

[3] Hays RD, Skootsky SA (2022). Patient experience with in-person and telehealth visits before and during the COVID-19 pandemic at a large integrated health system in the United States. J. Gen. Intern. Med..

[4] Ftouni R, AlJardali B, Hamdanieh M, Ftouni L, Salem N (2022). Challenges of telemedicine during the COVID-19 pandemic: a systematic review. BMC Medical Informatics and Decision Making..

[5] Liaw WR, Jetty A, Coffman M, Petterson S, Moore MA, Sridhar G (2019). Disconnected: a survey of users and nonusers of telehealth and their use of primary care. Journal of the American Medical Informatics Association..

[6] White SJ, Nguyen A, Cartmill JA (2022). Agency and the telephone: patient contributions to the clinical and interactional agendas in telehealth consultations. Patient Educ. Couns..

[7] Bombard Y, Baker GR, Orlando E, Fancott C, Bhatia P, Casalino S (2018). Engaging patients to improve quality of care: a systematic review. Implementation Science..

